# Evolutionary variation in the mechanics of fiddler crab claws

**DOI:** 10.1186/1471-2148-13-137

**Published:** 2013-07-15

**Authors:** Brook O Swanson, Matthew N George, Stuart P Anderson, John H Christy

**Affiliations:** 1 Department of Biology, Gonzaga University, 502 E Boone Ave, Spokane WA 99258, USA; 2 Smithsonian Tropical Research Institute, Apartado Postal 0843-03092, Balboa, Ancon, Republic of Panama

**Keywords:** Closing force, Cuticle damage, Structural damage, Signal efficiency, Trade-off

## Abstract

**Background:**

Fiddler crabs, genus *Uca*, are classic examples of how intense sexual selection can produce exaggerated male traits. Throughout the genus the enlarged “major” cheliped (claw) of the male fiddler crab is used both as a signal for attracting females and as a weapon for combat with other males. However, the morphology of the major claw is highly variable across the approximately 100 species within the genus. Here we address variation, scaling, and correlated evolution in the mechanics of the major claw by analyzing the morphology and mechanical properties of the claws of 21 species of fiddler crabs from the Pacific, Gulf and Atlantic coasts of the Americas.

**Results:**

We find that the mechanics that produce claw closing forces, the sizes of claws and the mechanical strength of the cuticle of claws are all highly variable across the genus. Most variables scale isometrically with body size across species but claw force production scales allometrically with body size. Using phylogenetically independent contrasts, we find that the force that a claw can potentially produce is positively correlated with the strength of the cuticle on the claw where forces are delivered in a fight. There is also a negative correlation between the force that a claw can potentially produce and the size of the claw corrected for the mass of the claw.

**Conclusions:**

These relationships suggest that there has been correlated evolution between force production and armoring, and that there is a tradeoff between claw mechanics for signaling and claw mechanics for fighting.

## Background

The evolution of exaggerated male secondary sex characteristics is frequently attributed to sexual selection either by female choice or combat among males [1,2]. To differentiate these two modes of selection, traits are separated into two groups based on their morphology and function. Morphological characters that are enlarged, conspicuously colored or shaped (e.g. peacock rump feathers), and are used primarily during courtship are ornaments; they are selected by female preferences that govern mate choice [3,4]. Morphological characters that are used as weapons are armaments; they are selected for their utility in intra-sexual combat [2,5].

**Figure 1:**
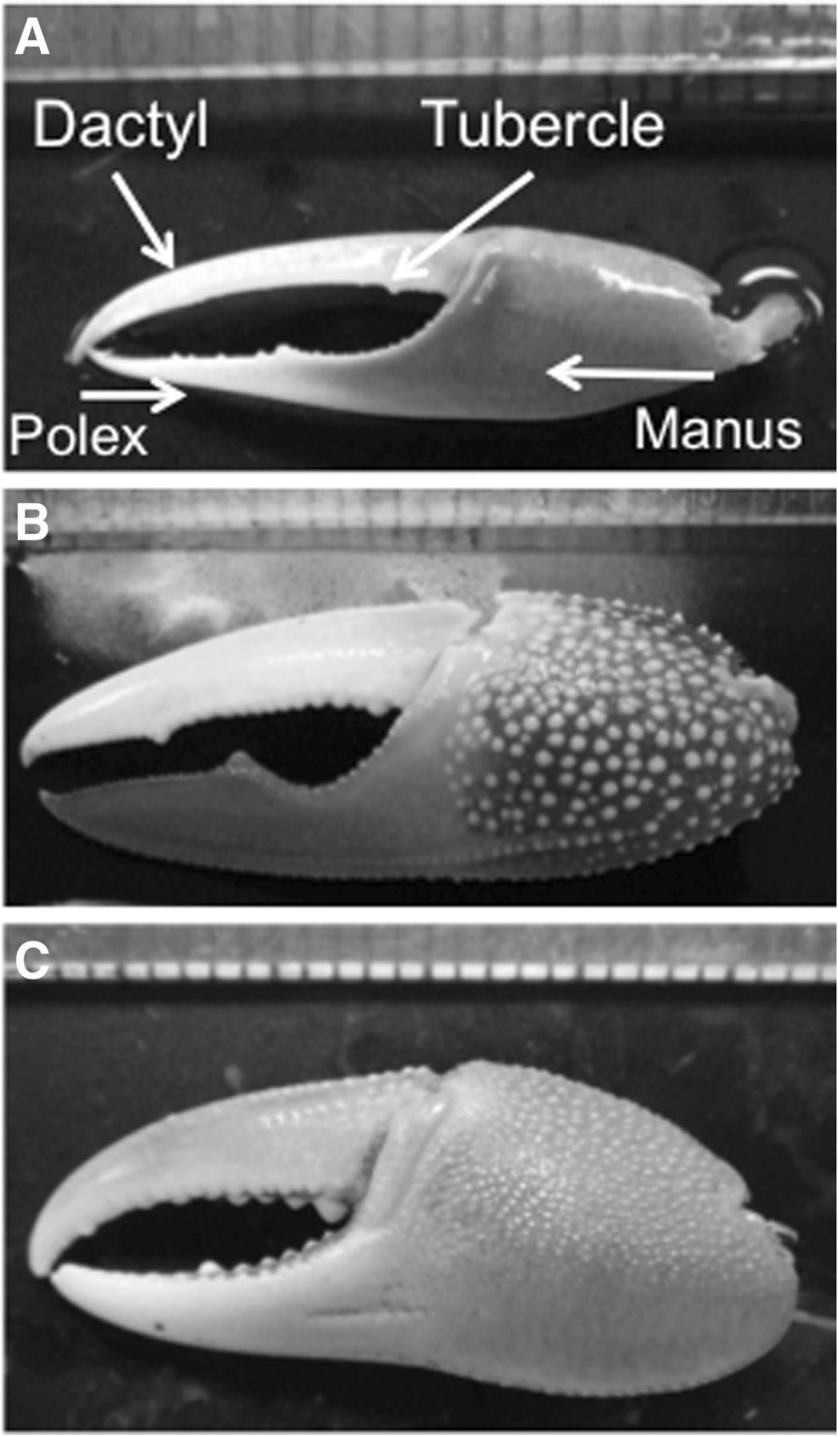
**Examples showing just a few of the divergent morphologies found in the fiddler crab major claw. (A)***Uca terpsichores* has an elongated claw with gracile polex and dactyl, **(B) ***Uca stylifera* is a large, basal species, **(C)***Uca argolicola* is a small species with a relatively short robust claw. During signaling, male crabs wave these claws in a species-specific pattern. During intra-sexual combat males interlock claws and apply force to a competitor’s manus using a dactyl or polex tubercle as indicated by arrows in frame **(A)**.

To complicate matters, many organisms have secondary sex characteristics that are both ornaments and armaments [2,5]. In most traits where both intra-sexual competition and inter-sexual selection are important, these forces act in concert, reinforcing the effects of both types of selection [2,4,6]. However, in fiddler crabs and several other species [6,7], the two mechanisms of sexual selection seem to be in opposition, favoring different features of the sexually selected traits [7]. In fiddler crabs (genus *Uca*), all of the approximately 100 species are sexually dimorphic. Females have two small claws and no visible asymmetry, whereas males have a single minor claw, with which they feed, and a hypertrophied major claw, which can constitute 1/3 – 2/3 of the total mass of an individual [8]. The shape, size, and coloration of the major claw are highly variable across species (Figure 1) [9]. Males use their major claw to court females, threaten males, and as a weapon when males fight. We suggest that the use of the major claw as both an ornament and an armament should select for different features of the claw [10,11]. To be the most effective sexual signal, the claw should be large, with a large surface area, yet be lightweight so that males can wave it at a low energetic cost. To be the most effective weapon, the claw should be relatively heavy, with a large closing muscle, a thick cuticle, and a relatively short dactyl and polex to maximize mechanical advantage.

Like many sexual ornaments, the major claw grows with positive allometry [12,13]. It is made conspicuous as an ornament by its coloration, reflectance [14], shape [15], and size [16]. Males of most species use their major claw in a variety of complex claw-waving displays that differ greatly across species [17-19]. Females have been shown to choose mates based on a complex set of species-specific criteria, which include, body or claw size [9,20-22], wave rate or height [17,23-25], burrow residency, and burrow quality [20,26-28]. Waves not only attract females to a male’s burrow for mating, but also ward off ‘wandering’ males that fight burrow residents for their burrows [8,18,29,30]. It is also possible that females select costly displays (vigorous waving with heavy claws) as a measure of male quality [4], however females will still select cheating males with regenerated claws and little combat ability [16]. The importance of the waving display led Huxley [12] to conclude that the major claw evolved primarily for signaling, with combat as an incidental secondary use, made possible by the increase in claw size.

**Figure 2:**
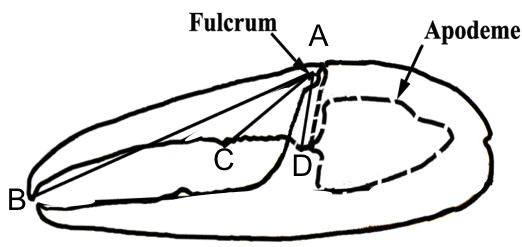
**Major claw outline of a male *Uca beebei*.** Measurements used in the study: **A** – **B**, out-lever length from the fulcrum to the claw tip; **A** – **C**, out-lever length from the fulcrum to the tubercle in the gape of the claw; **A** – **D**, in-lever length from the fulcrum to the apodeme insertion on the ventral portion of the dactyl (modified from Dennenmoser and Christy 2013).

Male fiddler crabs use combat to obtain and defend burrows and, when resident at a burrow, to displace neighboring males [31]. The form of combat varies in detail across species, but usually includes a sequence of ritualized behaviors, culminating in males interlocking and forcefully gripping their major claws, so that tubercles on the dactyl or polex of the claws apply force to the manus of the other crab (Figure 2) [8,10]. The closing forces males can produce with their major claw depend on the shape of the claw, and the size of the closing muscle [13]. Backwell et al. [16] found that crabs with more forceful claws and heavier cuticles tend to win fights with size-matched conspecifics. Therefore, it has been suggested that the claw demonstrates adaptations for use as a weapon [8-10,30], and in contrast to Huxley [12], it has been suggested that the major claw evolved primarily as an armament for male-male competition, with male–female signaling emerging as a byproduct of male-male agonistic interactions but see [2,18].

However, it is important to note that the claws of all fiddler crab species that have been studied must effectively perform both of these functions. For instance, an individual with purely signaling morphology would be at an energetic advantage in both development and display, but would not be able to defend territory and should have low fitness [16]. Alternatively, a crab with a shorter or excessively heavy claw, although successful in gaining and defending territory, would incur an energetic cost and may not be able to attract females, therefore having lower fitness [17,23-25]. Here we examine how a diverse set of fiddler crabs solve the problem of conflicting sexual selection on claw morphology. First, we examine variation across the clade and allometry of the mechanics of the claw across species. Second, we examine the relationship between claw mechanics and the mechanical properties of the cuticle. Third, we ask whether there is evidence of a tradeoff between mechanics that should maximize the claw’s effectiveness as a sexual signal and mechanics that should maximize the claw’s effectiveness as a weapon.

## Results

**Figure 3:**
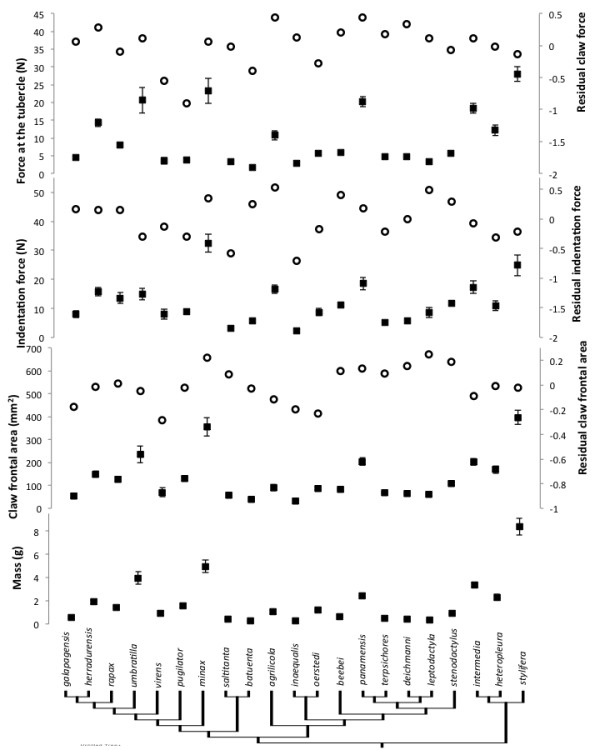
**Species averages (± 1 S.E.M.) of morphological and mechanical variables across the phylogeny of fiddler crabs.** Filled squares are the measured values, and open circles are corrected for body size: residuals of the OLS regression of the variable against body mass. Body mass, frontal area of the claw, an estimate of signal size, force required to puncture the cuticle, and force estimated at the claw tubercle are given (see text). Phylogeny is modified from Rosenberg, 2001. Branch lengths are arbitrary.

**Figure 4:**
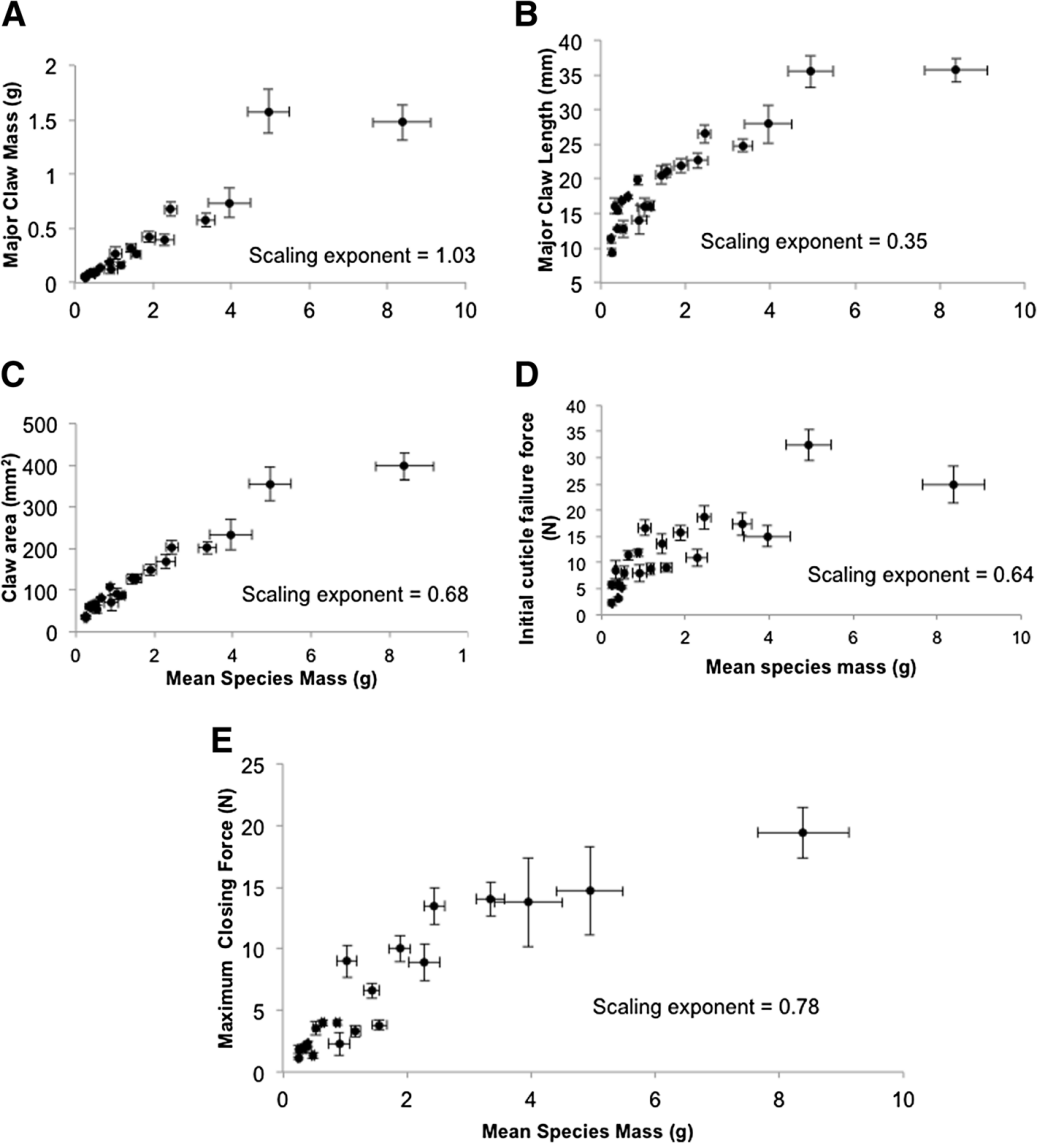
**Scaling of morphological and mechanical variables with crab body mass.** Each symbol represents a species average (± 1 S.E.M.) for both the variable in question and body mass. Scaling exponents are slopes of a regression of the logs of the mean values. **(A)** Mass of the major claw, **(B)** length of the major claw, **(C)** frontal area of the major claw, **(D)** Force required to puncture the manus cuticle, **(E)** Force estimated at the claw tubercle, calculated from the morphology of the claw, see text.

**Figure 5:**
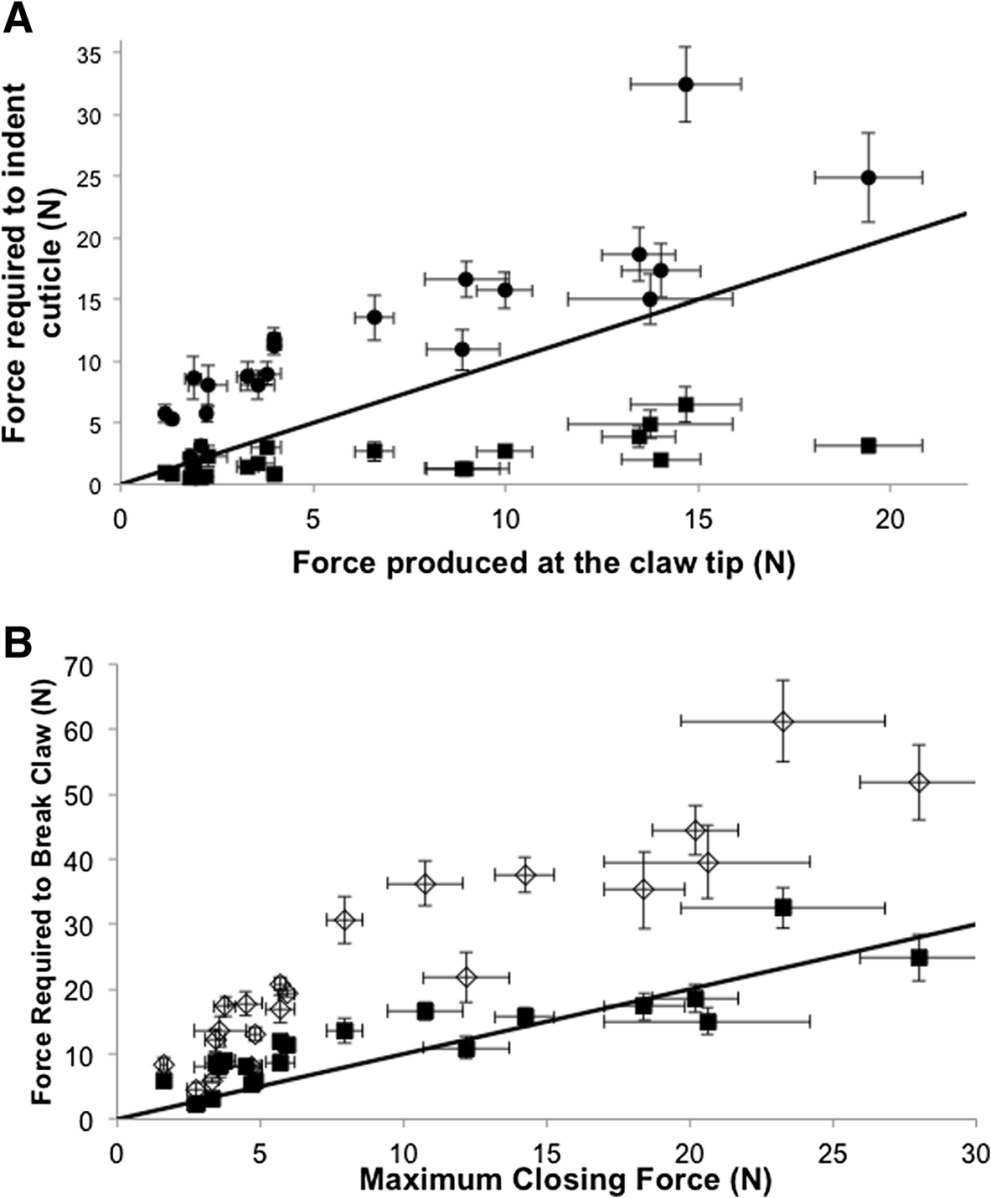
**The relationship between forces produced by the claw and resistance of the cuticle.** Each symbol represents a species average ± 1 S.E.M. **(A)** The relationship between the force estimated at the tip of the claw and the force required to indent the carapace (closed squares) and the force required to indent the manus (closed circles). The black line is an x=y relationship. Species averages that fall above the line (i.e. all of the manus values) are examples where the claw tip cannot produce enough force to puncture the cuticle. Species averages that fall below the line (i.e. all of the carapace values) can be punctured by the claw tip. **(B)** The relationship between force produced at the claw tubercle and the force required to indent (closed squares) and crack (open diamonds) the manus cuticle. Again, the black line is an x=y relationship. Indentation forces fall around the line and suggest that crabs can produce approximately enough force to indent the manus of a conspecific. However, crabs cannot produce enough force at the tubercle to crack the cuticle and cause catastrophic damage (i.e. these values fall above the x=y line).

**Figure 6:**
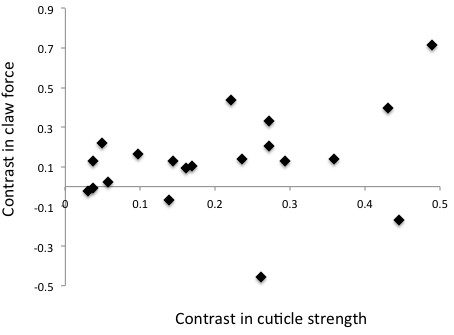
**The evolutionary relationship between claw force and armoring.** Symbols indicate the force required to cause cuticle indentation of the major claw plotted against the closing force of the major claw. All values are phylogenetically independent contrasts of weight-normalized species averages. Linear fits of standardized contrasts are constrained to pass through the origin and there is a significant positive relationship (r = 0.57, F=8.97, d.f.=19, p<0.05) suggesting that crabs with forceful claws also tend to be heavily armored, even when taking into account size and phylogenetic relatedness.

**Figure 7:**
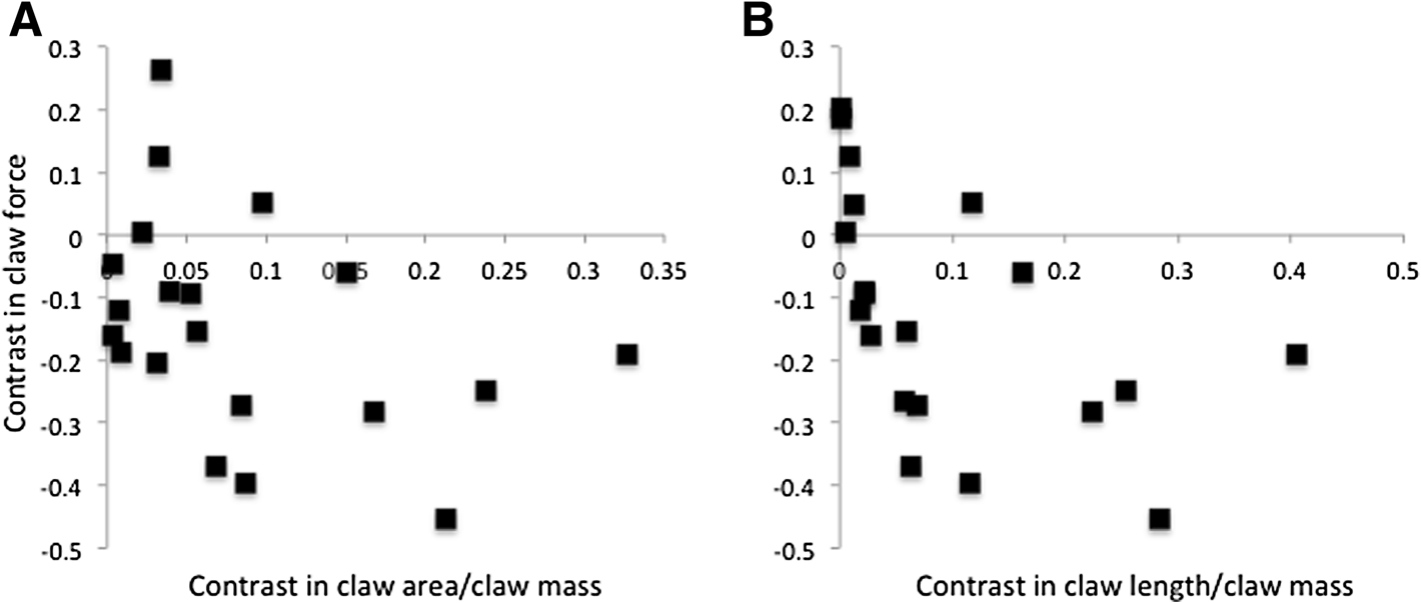
**The evolutionary relationship between claw force and signaling morphology. A)** Symbols are claw frontal area divided by claw mass, plotted against the estimate of major claw closing force at the tubercle. **B)** Symbols are claw length divided by claw mass, plotted against the estimate of major claw closing force at the tubercle. All values are phylogenetically independent contrasts of weight-normalized species averages. Linear fits of standardized contrasts are constrained to pass through the origin and there is a significant negative relationship between these variables (claw area: r=-0.67, F=15.3, d.f.=19, p<0.05; claw length: r=-0.69, F=17.69, d.f.=19, p<0.05). As species can produce relatively larger closing forces, the major claw’s efficiency as a signal decreases, implying that there is an evolutionary trade-off between morphology that should maximize the major claw’s functionality as a weapon and morphology that should maximize the major claw’s functionality as a signal.

We found considerable morphological variation across the fiddler crab phylogeny (Figures 1, 2, 3). Most of the variables measured seem to scale isometrically with mass (Figure 4). For instance, the values of the exponents of the regressions of the mass of the major claw (1.03), the length of the claw (0.35), and the frontal area of the claw (0.68), on body mass all are exactly as expected (1, 1/3, and 2/3 respectively; Figure 4). Cuticular resistance scales at approximately 2/3 power of body mass although there is a large amount of variation in this value, with an r^2^ of only 0.78. The scaling exponent of the force produced at the tubercle of the claw was 0.78, higher than the expected value of 2/3 for scaling of the muscle cross sectional area (Figure 4). When the force produced by the claw is compared to the ability of the cuticle to resist puncture, we find that within a species, the claw tip should be able to produce enough force to puncture the carapace, but not the manus. Furthermore, we find that crabs should be able to produce approximately enough force at the claw tubercles to puncture the manus cuticle of a size-matched conspecific, but not enough force to cause the cuticle to structurally fail (Figure 5). When claw force and claw resistance values are adjusted for size (residuals of the OLS regression of ln transformed values on ln body mass) there is a positive correlation (r=0.615, d.f.=19, p<0.05). When values are further corrected for phylogeny (standardized independent contrasts), we find that there is still a positive correlation between claw force and the force required to puncture the manus cuticle (r = 0.57, F=8.97, d.f.=19, p<0.05; Figure 6). Finally, there is a significant negative correlation between force produced at the tubercle (mechanics that should be good for combat) and both weight specific frontal area of the claw and weight specific claw length (morphologies that should provide a large signal at low waving cost) both for size corrected values (claw area: r=-0.44, d.f.=19, p<0.05; claw length: r=-0.48, d.f.=19, p<0.05), and when using size-corrected standardized contrasts (claw area: r=-0.67, F=15.3, d.f.=19, p<0.05; claw length: r=-0.69, F=17.69, d.f.=19, p<0.05; Figure 7).

## Discussion

### Mechanical variation

We found that the mechanics of the major claw are highly variable across species. This has been observed by others [8-10] and it is known that claw strength is tightly, positively correlated with size [13]. However, even when the effect of size is removed we found variation of more than two-fold in claw force potential across species (Figure 3). This is due to variation in apodeme area (a proxy for muscle physiological cross sectional area) and morphology of the dactyl (Figure 2).

We also found that the force required to fracture the cuticle of the claw is variable, even with the effect of size removed (Figure 3). This variation probably results from differences in the material properties, thickness and shape of the cuticle. For instance, crabs may produce resistant claws by either making their cuticular material stronger or by producing a thicker cuticle. We do not know how the material properties vary across species, and this will be a focus of future work. We do know that body size corrected claw mass is correlated with resistance to puncture, suggesting that crabs increase resistance by increasing cuticle thickness (not shown).

### Allometry

Although mechanical properties of the claw are correlated with body size, there is precedent to suggest that sexually selected traits should scale allometrically [12,13,32]. However, Bounduriansky [33] suggests that this purported pattern is due to sampling bias and argues that under varying selective circumstances there should be different allometric patterns. We find no evidence that simple measurements of claw size are positively allometric across species (Figure 4). This contrasts with pronounced positive developmental allometry within some species of *Uca*[12,13], and suggests that there may be different patterns of selection acting on different species [33]. Most of the variation in claw mechanics across species seems to be associated with isometric scaling. The only exception is claw force production, which may suggest that this trait is under intense sexual selection across the clade. Alternatively, claw size, but not force, may be constrained. Levinton and Judge [13] noted that claw length seems to be constrained by carapace width so that claws do not extend beyond the contralateral edge of the carapace. When the crab rotates to enter its burrow the tip of the claw must go with the body (males hold their claws “folded” across their front when they enter their burrow). Hence, claw length at full adult size would scale isometrically with body width across species. The forces a claw can produce do not seem to be under this constraint.

### Claw force and cuticle strength

When we compare the average force produced by the claws of a species to the resistance of the cuticle of that species to puncture, we find that all species should be able to break the carapace but not the manus of conspecifics with their claw tips (Figure 5). This makes sense in light of the ritualized combat seen in fiddler crabs, where only the manus (and not the carapace, which would cause serious injury) is gripped during aggressive interactions [8-10]. Like other species of crabs, male fiddler crabs grip each other’s major claw manus with the tubercles within the gape of their own major claw. The mechanical advantage at the tubercles within the gape of fiddler crab claws exceeds that at the tip of the claw. This seems to result frequently in surface indents, or small cuticular failures at the point where the tubercles contact the manus [10]. We found that the forces at these tubercles and the forces required to cause an indentation in the cuticle of the manus of the claw were very similar across all species (Figure 5). This may reflect an optimization between force production and cuticular strength where the forces are received in a fight. We assume that increasing either of these variables comes at a cost. In fact, Murai et al. [34] found that experimentally increasing the mass of the claw not only increases the waving cost, but also decreases the wave height, an important component of female choice. It appears that crabs produce cuticles just strong enough to resist damage from most of their conspecifics, and have claws strong enough to cause slight surface damage to opponent’s claws. This provides a mechanical explanation for why males usually choose to fight males their size or slightly smaller [31], and why larger crabs tend to win [35]. This could also help explain why crabs with regenerated and presumably weaker claws lose in fights [16].

As expected, larger crab species produce more force and are more resistant to cuticular damage. However, when the effects of size and phylogenetic relatedness are removed, there is still a significant positive relationship between force production and resistance to puncture (Figure 6). Hence, species with especially powerful claws also tend have especially armored claws, suggesting that these traits have evolved in concert. It also suggests that selection on claw force or cuticle resistance varies across the *Uca* clade.

### A possible tradeoff between signaling and force

One of the most intriguing features of the major claw is that it functions both as a semaphore and a weapon. The mechanical properties one expects of semaphores or signaling flags are very different from those one expects of weapons. We suggest that flags (visual signals) should be large and lightweight to minimize the cost of display. It has been shown that claw size, and especially length, are selected by females [34]. However, it has also been shown that male fiddler crabs incur significant energetic costs displaying with their massive claw [36]. Weapons, on the other hand, should be relatively short to maximize mechanical advantage and heavy to maximize cuticle thickness and muscle cross sectional area [11]. Indeed, we found an inverse relationship between claw force production and both the frontal area of the claw divided by claw mass and the length of the claw divided by claw mass (with the effects of body size and phylogenetic relatedness removed; Figure 7). There are of course alternative explanations for this pattern. For instance, both of these variables may be driven by some unmeasured variable. We also do not know the actual energetic costs of waving in any of the species studied here. There are also compensation mechanisms that allow species to have large displays and strong claws. For instance, *U. terpsichores* has an above average claw force and an above average mass specific claw area (Figure 3) [10]. However, in general we expect that species in which combat is more important in determining male success should have claws that mechanically are more like weapons, and that species in which signaling is more important should have claws that are mechanically more like flags. For instance, *U. heteropleura* and *U. saltitanta* both have claws that are mechanically weak and seem to have exceptionally intense waving behaviors [[9]; personal observations].

Within the more derived clades of fiddler crabs, we see several examples of the evolution of more forceful claws that can produce and resist much higher forces than the basal clade. However, this seems to come at a cost in that these claws are relatively smaller and are heavier. We hypothesize that these changes in claw structure are likely due to changes in social structure or courtship behavior. However, this remains to be tested experimentally. There are several examples where strong or resistant claws are found on species where the importance of waving seems to be reduced. For instance, *U. argillicola* is a small species with an unusually powerful claw that has never been observed to wave. *U. leptodactyla* has strong and resistant claws that seem to be waved infrequently. *U. stenodactylus* has a resistant claw and seems to have a mating system where female choice has reduced importance [9; personal observations]. With selection for signal function relaxed, incidental use of these claws for defense against predators might further accentuate mechanical features that make them good weapons. Unfortunately, the details of mate choice and aggressive interactions have not been well-studied in most *Uca* species. We suggest that our results should lead to more detailed examinations of these behaviors to see if our predictions hold. Hunt et al. [6] suggest that when selective forces act in opposition, variation in the timing, strength and direction of selection can produce a range of possible outcomes. This seems to be the case with fiddler crabs, where different morphologies may have been produced by variations in the details of the selective regime in each species.

## Conclusion

Fiddler crabs are one of the classic examples of female choice producing an exaggerated, male trait, the major claw [9,12]. However, our analysis suggests that the evolution of the major claw has been much more complex and interesting than if it had been selected only for signaling. The evolutionary challenge for a fiddler crab is to produce a conspicuous semaphore that retains its utility as a formidable weapon (the problem of how to design a “beautiful weapon”; [10]). There is significant variation in the mechanics of claws across species, both in force production and armoring. Within this variation, we find evidence of an evolutionary tradeoff, where different species solve this central problem of conflicting selection in different ways. Conflicting selection may maintain genetic and morphological diversity in species [7], and we suggest that it may also drive the evolution of morphological and functional diversity within a clade.

## Methods

### (A) sample collection and morphological measurements

Specimens of 21 fiddler crab species (*Uca*) were obtained from field sites in Panama and from biological supply companies as indicated in Table 1. Only adult males were used in this study. Sample sizes varied, with the majority of species being represented by at least 15 individuals (Table 1).

**Table 1:** List of species sampled and collection sites for the crabs used in this study

**Species**	**N**	**Collection site**	**Mass (g)**	**Mass (g)**	**Carapace width**
			total	major claw	(mm)
*Uca argillicola*	14	Diablo Heights, Panama	1.04 ± 0.59	0.27 ±0.20	12.62 ± 2.44
*Uca batuenta*	10	Rodman, Panama	0.25 ±0.07	0.05 ±0.02	7.35 ± 0.68
*Uca beebei*	20	Rodman, Panama	0.64 ± 0.11	0.14 ± 0.03	10.37 ±0.61
*Uca deichmanni*	20	Rodman, Panama	0.41 ± 0.09	0.09 ±0.03	9.03 ± 0.74
*Uca galapagensis*	11	Diablo Heights, Panama	0.54 ± 0.2	0.10 ±0.07	10.35 ±1.25
*Uca herradurensis*	25	Diablo Heights, Panama	1.89 ± 0.66	0.42 ±0.18	16.14 ± 1.89
*Uca heteropleura*	16	Rodman, Panama	2.28 ± 1.01	0.39 ± 0.2	17.64 ± 2.28
*Uca inaequalis*	13	Rodman, Panama	0.26 ± 0.08	0.05 ± 0.02	7.37 ± 0.70
*Uca intermedia*	7	Rodman, Panama	3.35 ± 0.61	0.58 ± 0.16	20.09 ± 1.28
*Uca leptodactyla*	8	Galeta, Panama	0.34 ± 0.09	0.08 ± 0.03	8.76 ± 0.70
*Uca minax*	15	Commercial vendor	4.97 ±2.12	1.50 ± 0.84	22.88 ±3.35
*Uca oerstedi*	14	Rodman, Panama	1.17 ± 0.30	0.17 ± 0.06	12.46 ± 1.00
*Uca panamensis*	15	Naos, Panama	2.45 ± 0.63	0.67 ± 0.25	16.82 ± 1.44
*Uca pugilator*	13	Panacea, Florida, USA	1.55 ± 0.45	0.26 ± 0.09	14.84 ± 1.43
*Uca rapax*	15	Galeta, Panama	1.43 ±0.48	0.32 ± 0.15	14.67 ± 1.46
*Uca saltitanta*	15	Rodman, Panama	0.39 ± 0.07	0.09 ± 0.02	8.08 ± 0.54
*Uca stenodactylus*	20	Rodman, Panama	0.88 ± 0.15	0.19 ± 0.04	11.65 ± 0.60
*Uca stylifera*	14	Rodman, Panama	8.38 ± 2.77	1.48 ± 0.62	26.04 ± 2.97
*Uca terpsichores*	20	Rodman, Panama	0.49 ± 0.07	0.08 ± 0.02	9.93 ± 0.54
*Uca umbratila*	11	Diablo Heights, Panama	3.95 ± 1.83	0.74 ± 0.47	19.38 ± 3.02
*Uca virens*	12	Panacea, Florida, USA	0.91 ± 0.62	0.12 ± 0.14	12.46 ± 2.50

The total weight, claw weight, and carapace width was averaged within each species and is given with its corresponding standard deviation.

Individuals were removed from their burrows and measurements were taken in the field. Carapace breadth (mm) was measured with digital calipers (Mitutoyo, CD-6”CSX). Total mass of each individual (g) was determined with a digital scale (Ohaus, Scout Pro). For each individual, pressure was applied to both the anterior and posterior surfaces of the merus, resulting in the autonomy of the major cheliped. We immediately released these males close to where they were collected. The mass of the major claw was recorded (g) without the carpus or merus attached. Removed limbs were placed in labeled plastic bags filled with seawater and transported on ice to the Smithsonian Tropical Research Institution’s Naos Laboratory, Panama. Five whole individuals of each species were also collected for carapace analysis. The averages with standard deviations for carapace breadth, total weight, and major claw weight of each species collected are presented in Table 1.

All samples were analyzed within 10 hours of collection. A photo was taken of each major claw with a Pentax Optio W60 camera. Major claw length was the distance from the base of the juncture between carpus and manus and the tip of the pollex. The dactyl height (in-lever length) was measured from the fulcrum to the insertion of the closer apodeme (Figure 2: [13]. Dactyl length (out-lever length) was split into two measurements due to variation of claw morphologies across species. The distance from the fulcrum to the dactyl tip and to the innermost large tubercle was recorded (Figure 2). All measurements were taken using ImageJ (http://rsbweb.nih.gov/ij/).

### (b) resistance to puncture

Within the genus *Uca*, intra-specific fighting between males can escalate into high intensity combat where two individual’s major claws interlock while each crab grips the other [8]. In this position, tubercles on the dactyl come into contact with the anterior manus of the opponent [10]. In light of this behavior, we designed a macro-scale puncture test to measure how the manus of the major claw reacts to point forces. Using an INSTRON Inspec 2200 portable bench-top materials tester (INSTRON, Norwood, MA, USA), the anterior surface of the manus, where claw-closing forces are delivered, was punctured using a conical tip that emulated the shape of the tubercles. This produced a force/displacement curve. The first peak of each curve was interpreted as the force at which the cuticle of the major claw began to fail. This caused a small, circular indentation in the cuticle, like those often seen on the claws of crabs in the field [10, personal observation]. The tip was advanced further and force increased until the manus cuticle structurally failed (cracked) and force dropped. This procedure was repeated on three locations on the carapace (dorsal surface) of a subset of five individuals per species.

### (c) estimation of closing force

We used morphological measurements to estimate the closing force of the major claw. Crabs have a bipinnate closer muscle in the manus of each claw that is attached to a chitinous disk, the closer apodeme (Figure 2; [37]). Contraction of the closer muscle pulls on this apodeme from both sides. The apodeme is connected to the base of the dactyl and closer muscle contraction produces a moment (force of the muscle*height of the dactyl, Figure 2) and rotates the dactyl [11,37,38]. Force is then exerted at the tip of the claw or the tubercles within the gape of the claw. Closing force is therefore a function of the stress produced per cross-sectional area of muscle, the cross-sectional area of the closer muscle (MCA, which is equal to 2* apodeme area) [37,38] and the mechanical advantage of the lever arm (MA, which is the ratio of the dactyl height (the “in lever,” measured from fulcrum to the point of connection with apodeme) to the length of the lever arm (the “out lever,” measured from the fulcrum to the point force is applied); Figure 2; [39-41].

Two MA values were defined when appropriate, one using the out-lever length to the tip of the claw and the other to the most proximal tubercle in the gape of the claw. Apodeme area was determined by photographing the exposed apodeme and measuring its area using ImageJ (http://rsbweb.nih.gov/ij/).

In order to estimate the actual closing force that each species is able to produce, force per unit of muscle cross-sectional area was determined by measuring closing force directly in *Uca minax*. We attached hooks to the Inspec 2200 portable bench-top materials tester. Wire was wrapped around both the dactyl and pollex of five individuals and the maximum closing force (F_max_) was recorded for each individual. Claws were then removed and the mechanical advantage (MA), the apodeme area (equivalent to 1/2 MCA) and average angle of pinnation of the muscle fibers when stretched and relaxed (α) was determined using ImageJ. Stress generated per unit of muscle cross-sectional area (σ) was then determined using the following equation for each individual:

$$\begin{array}{c} \mathrm{Stress}\;\mathrm{generated}\;\mathrm{per}\;\mathrm{unit}\;\mathrm{of}\;\mathrm{muscle}\;\\ \mathrm{cross}-\mathrm{sectional}\;\mathrm{area}\left(\sigma \right)\;=F_{\max}/\mathrm{MA}*\mathrm{MCA}*\sin\;2\alpha . \end{array}$$

Stress generated per unit of muscle cross-sectional area (σ) was averaged for the five individuals tested and the resulting σ_avg._ was treated as a constant across species. There may be differences across species in muscle physiology or sarcomere length that may produce differences in force per cross sectional area. However, this method allowed us to address how claw morphological variation should affect force production. The closing force of the major claw was then estimated for each individual using the following equation:

$$\mathrm{Maximum}\;\mathrm{Closing}\;\mathrm{Force}\left(N\right)=\mathrm{MA}*\mathrm{MCA}*\sigma_{\mathrm{avg}}.$$

### (d) statistical analysis

When comparing morphological characters among species, the relative size and the phylogenetic history of each species can drive correlations [42,43]. To correct for differences in size, we took the ln of the mean of each morphological character of each species and plotted that value against the ln of the mean of the body mass of each species. Residuals of the OLS regression were calculated giving values of each morphological measurement that were corrected for mass and normalized for each species [44]. Phylogenetically independent contrasts of the species values (mass corrected and normalized) of each morphological measurement were determined using the PDAP package for Mesquite [45,46] and the strict consensus of 8 most-parsimonious trees of the genus from Rosenburg [47]. A series of different branch lengths were tried in the analysis. Setting all branch lengths equal to one minimized the relationship between the absolute values of the standardized independent contrasts and their standard deviations [43]. We therefore set all branch lengths equal to one when calculating phylogenetically independent contrasts for subsequent analysis. When there is no phylogenetic signal in the data, non-phylogenetically corrected statistics are more appropriate. However, univariant estimates of phylogenetic signal can fail to detect it in some situations [48]. Therefore, correlations between variables were also explored without phylogenetic correction.

We estimated the efficiency of the major claw as a signal two ways 1) as the ratio of the length of the claw to the mass of the claw, and 2) as the ratio of the surface area of the claw (its visually apparent size) to the mass of this structure. Large values of these ratios indicate a large claw of small mass that should require relatively little energy to wave.

## Competing interests

The authors have no financial or non-financial competing interests associated with this research or the publication of this manuscript.

## Authors’ contributions

BS conceived of the study, collected data, analyzed data and wrote the manuscript. MG was involved in experimental design, collected and analyzed data and wrote the manuscript. SA was involved in experimental design and collected and analyzed data. JC conceived of the study, collected data and significantly revised the manuscript. All authors read and approved the final manuscript.
